# Technical note: An automated document verification tool in radiation oncology EMR: Application for LDR prostate brachytherapy

**DOI:** 10.1002/acm2.14466

**Published:** 2024-07-30

**Authors:** Junliang Xu, Baoshe Zhang, Mariana Guerrero, Chaitanya Kalavagunta, Shifeng Chen, Huijun Xu

**Affiliations:** ^1^ Department of Radiation Oncology University of Maryland School of Medicine Baltimore Maryland USA

**Keywords:** automated chart check, LDR brachytherapy, periodic audits, prostate cancer, script

## Abstract

Purpose

This study aims to illustrate how a script‐based automated tool can efficiently verify documentation for LDR prostate brachytherapy. Methods and Materials: An in‐house Python‐scripts‐based tool was developed to automatically verify the specific checklists, aligned with our institutional practice guidelines for prostate seed implants (PSI). The scripts, compatible with our radiation oncology information system, could be executed with an optional web‐based middleware to access and evaluate Aria documents. Optimized based on data from the previous 400 patients, the automated tool was applied to a random cohort of 50 LDR patients. It evaluated the adequacy of specific EMR documents by performing checks for data completeness, consistency, and allowable value range. We analyzed the efficiency of using this tool against conventional manual checks in two LDR processes: seed ordering and monthly audits for our PSI programs. Results: The automated tool effectively performed chart checks on the involved PSI documents. Human errors, such as typos and inconsistent information, were identified in 7 out of 50 patients during the seed ordering process and in 2 out of 50 patients during the monthly audit. Meanwhile, this automation reduced the majority of manual chart‐checking time by an average of 5 and 10 min per patient for these processes, respectively. The anticipated efficiency gains will continue to accrue as more check items are digitalized and assessable to the scripts. Conclusions: The implementation of an automated tool tailored for LDR prostate brachytherapy has demonstrated its efficiency benefits. Such an approach can help other clinics substantially enhance routine chart checks, periodic audits, and other applications in similar clinical settings.

## 1 INTRODUCTION

Low‐dose‐rate (LDR) brachytherapy, using implanted radioactive sources in the prostate, offers targeted radiation with steeper dose gradients, and therefore reduces complications compared to external beam radiotherapy (EBRT). Evidence^1^, ^2^ from five non‐randomized prostate cohort studies found LDR brachytherapy as effective as high‐dose‐rate (HDR) brachytherapy alone, but more beneficial when combined with EBRT. Proper documentation ensures that LDR procedure is meticulously recorded, monitored, and reviewed, allowing medical professionals to optimize their clinical practice to meet the standards set forth by the American Association of Physicists in Medicine (AAPM)^3^, ^4^, ^5^ and the American Brachytherapy Society (ABS).^6^

The debate over whether automation can completely replace manual documentation checks is ongoing.^7^ Nonetheless, it is clear that automation significantly boosts workflow efficiency, reduces human errors and burnout, and strengthens patient safety.^8^ With the shift from paper to digital patient documentation, numerous in‐house and commercial automation tools have emerged, primarily tailored for EBRT^9^, ^10^, ^11^, ^12^ and HDR brachytherapy.^13^, ^14^, ^15^, ^16^ The integration of automated documentation verification tools into LDR brachytherapy shows promise in offering Radiation Oncology practitioners a similar spectrum of advantages. However, unlike its counterparts in EBRT and HDR brachytherapy, computer automation for LDR brachytherapy documentation and chart checks remains largely unexplored.

This work addresses the automation of the chart check process for LDR prostate brachytherapy documentation verification by implementing in‐house Python scripts. The scripts were developed to be compatible with our setting of LDR brachytherapy Electronic Medical Record (EMR) system. To our best knowledge, this is the first study that proposes and evaluates an automatic documentation verification tool for LDR brachytherapy.

## 2 METHODS

### 2.1 LDR prostate brachytherapy procedure and documentation framework

An operational checklist was meticulously devised following established institutional protocols for LDR prostate brachytherapy in our institution. The documentation process predominantly adopts an electronic framework. Varian Aria (Varian, Palo Alto, CA, USA) is the radiation oncology information system (RO‐IS)‐based system. A suite of Microsoft Word templates within the Aria EMR system serve as the foundation for this electronic documentation.

Our prostate seed implant (PSI) process is briefly described here. A volume study based on a computed tomography (CT) scan was performed by a physician to assess prostate size and pubic arch interference. Results are transcribed into a prescription document detailing treatment objectives, dose, isotope potency, prostate stage, and historical context. Seeds are ordered by a physicist, with seed assay and review logged manually in the hot lab. Loose seed implantation is operated by the physician using ultrasound guidance, followed by real‐time planning and documentation by the physicist. End‐of‐treatment (EOT) documentation is completed by the physician, while a quality checklist and a survey report are filled out by the physicist. The post‐treatment planning for quality assurance is initiated weeks later, with the plan document uploaded into Aria EMR.

Table 1 lists the five relevant ARIA documents with the number of items checked in each of them. A total of 133 data points need to be examined for a comprehensive review of the patient's prescription, volume study report, quality checklist, survey report, and EOT documents.

**TABLE 1:** Five LDR prostate brachytherapy Aria documents with their numbers and examples of the check items that can be automatically checked by our in‐house scripts.

PSI document	No. of items checked	Examples of crucial checks
Prescription	20	Dose, definitive or boost treatment, prostate volume, seeds to be ordered versus nomogram
Volume report	16	Prostate volume, imaging modality against prescription, Pubic Arch Interference
Quality checklist	34	time‐out checklist, implant information (no. of needles/sources, cystoscopy)
Survey report	27	Radiation survey around patient (surface and 1 m from multiple anatomical locations) and in PECU
End of treatment	36	Operative note against quality checklist and survey report

### 2.2 In‐house word document chart check scripts: development and capabilities

**FIGURE 1:**
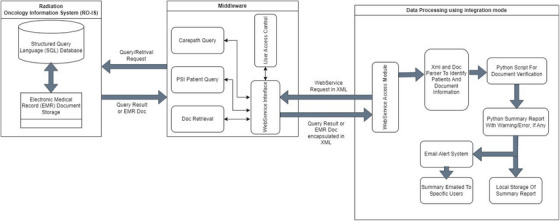
Infrastructure of the integration mode for our in‐house scripts to be integrated into RO‐IS Aria system to perform documentation verification for LDR PSI patient. From the middleware layer, it starts with identifying patients with active Care Paths status in Aria. It then extracts and parses EMR documents from the RO‐IS database for chart checking. The final step is generating summary reports that highlight practice variance. These reports can be saved in a specific directory or sent via email to the relevant users.

An in‐house tool composed of a series of Python (v3.11) scripts was developed to automatically check our EMR documents for LDR prostate brachytherapy. Our tool is compatible with two commonly used document formats, Word and PDF. The scripts can be executed in two modes: standalone and integration. Figure 1 provides an overview of how the scripts seamlessly integrate with the Aria EMR system through a middleware layer, which ensures data protection and security. In standalone mode, users can manually initiate operations of specific scripts. Integration mode enables an automated workflow, which begins with identifying a patient list based on their status of Care Paths, a communication system in Aria. It then proceeds to extract and autonomously parse specific EMR documents from the RO‐IS database to conduct the chart check process. Both modes generate summary reports highlighting practice variances. These reports can be saved in a designated directory, and in integration mode, they can be emailed to the relevant users.

Our automated documentation verification tool employs a multi‐layered approach: 1. Data completeness check ensures all mandatory fields within relevant EMR documents are filled in and meet expectations, such as verifying the “source activity” is filled in as 0.36 mCi for our practice. 2. Consistency check compares data points across different documents to identify any discrepancies that might indicate errors. For instance, it compares implanted seed numbers in the quality checklist and post‐PSI survey reports to match those in the plan report. If discrepancies arise, a completeness check is triggered to the document for explanation. 3. Allowable value range check confirms that specific data points fall within acceptable limits established by institutional protocols or clinical guidelines. For example, it verifies the seed quantities manually entered by physician against our institutional nomogram (Table 2) and implements a tolerance limit of ±5. If the manual number exceeds this nomogram by more than 5, a warning message prompts physicist verification. Conversely, if the manual number is lower by more than 5, an error message is issued for immediate attention and investigation.

**TABLE 2:** Our institutional nomogram as a guideline for PSI seed ordering.

Average number of seeds required (I‐125, 0.36 mCi per seed)		
Prostate (cm^3^)	110 Gy	145 Gy
10	31	43
15	38	50
20	45	57
25	52	64
30	59	71
35	66	78
40	73	85
45	80	92
50	87	99
55	93	106
60	100	113
65	107	120
70	114	127
75	121	134
80	128	141

To validate our tool, we created a series of test cases based on variances of the previous 400 PSI patients within our institution. These cases intentionally introduced errors/discrepancies for false negatives (missing errors) and included data that should not trigger any warning/error for false positives (incorrectly flagging items). Following this validation, the tool was refined and optimized. The scripts skip all the clipped figures or uploaded screenshots in the documents due to a limitation in interpreting images.

### 2.3 Patient selection, document analysis, and scripts application

A random cohort of 50 patients to get LDR prostate brachytherapy treatment utilizing I‐125 seeds implantation was selected for the evaluation of our automated scripts. These patients were prescribed with a dose of either 145 Gy for definitive treatment or 110 Gy for boost treatment following an EBRT with a dose of 45 Gy to the whole pelvis.

We highlight the application of our scripts in two key domains: seed ordering and monthly audits for our PSI program. To provide insights into the advantages of automation over conventional manual workflow, we assessed the error‐mitigating potential and time‐saving attributes achieved in these 50 patient cases. Two co‐authors recorded manual check time with and without the scripts to calculate the time saved by using the scripts.

## 3 RESULTS

### 3.1 Seed order automation

**FIGURE 2:**
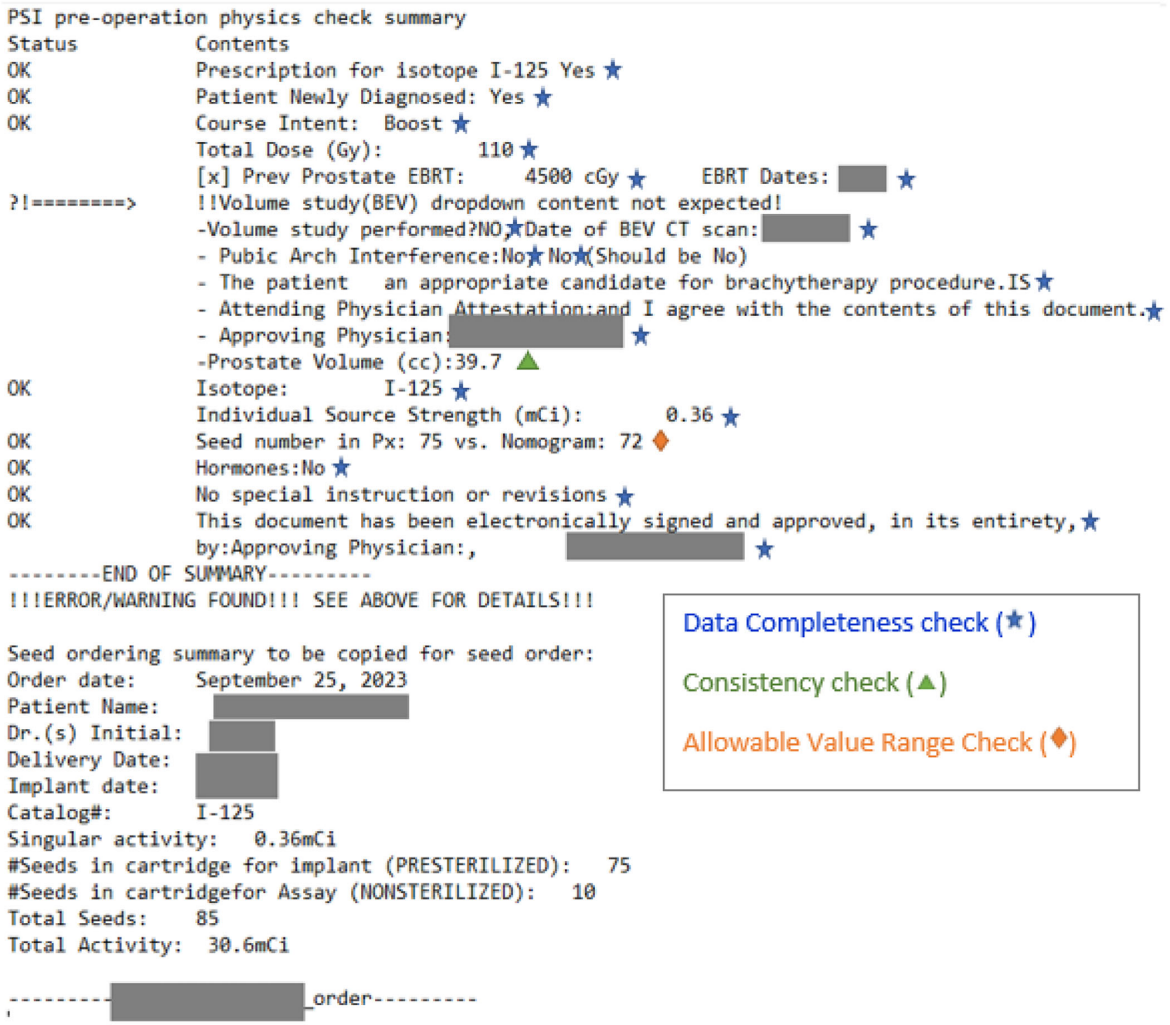
The summary of seed ordering check and order information generated by our scripts. Certain details have been withheld for privacy protection. The first half of this figure represents a sample seed ordering check report with 20 automatic checks performed on a single patient. Three types of checks—data completeness check, consistency check, and allowable value range check—have been illustrated with different symbols. We highlight a warning message of inconsistent prostate volume in the prescription versus the volume study report.

**FIGURE 3:**
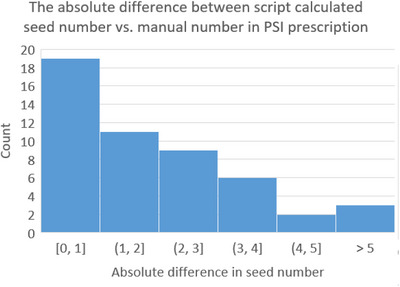
Histogram of the absolute difference between manual versus scripts calculated seed numbers. Because we consistently order seed numbers in increments of 5, a tolerance difference of 5 or less between manual numbers in the prescription document versus scripts‐calculated numbers is applied here. In this context, three out of 50 patients exhibit an absolute difference exceeding the defined tolerance, with the manually recorded number exceeding the script calculation. These particular cases trigger a highlighted warning message for further attention.

Seed order automation significantly enhances data accuracy, integrity and efficiency in the clinical workflow of LDR brachytherapy. The scripts generate detailed summary reports (Figure 2), highlighting critical data points such as unexpected dropdown content in volume studies for further review. This automation reduces typographical errors by providing precise information directly to seed vendors. In practice, the tool identified various issues, including typos, inconsistencies, and missing information. Additionally, the scripts flagged instances where seed calculations exceeded predefined tolerances, prompting warnings for closer inspection (Figure 3). For 7 out of 50 LDR prostate patients, the scripts detected typographical errors, information inconsistency, and incompleteness, including missing details in attestation, prior treatment history/dosage information, and discrepancies in the prostate volume specified in the volume study versus prescription documents. Three out of 50 patients exceeded the defined tolerance in seed number difference between manual and script calculations, all overestimated, triggering warning messages for closer examination. The automation tool streamlined the chart‐checking process for seed orders, saving over 5 min per patient on average.

### 3.2 Monthly audit automation:

Monthly audit automation validates a wide array of documents within clinical operations. Table 3 furnishes a detailed breakdown of document validations that are amenable to automation within our current clinical operational framework. Roughly half of the items under scrutiny in our institution have transitioned to digital formats, while the remainder persists in logbooks or physical binders. Our scripts facilitate digital documentation checks, thereby reducing the manual check time required for patient audits. For 50 LDR prostate patients, the tool achieves an average time saving of 10 min per audit, with the potential for even greater efficiency as more documentation is digitized. The scripts flagged two errors in LDR prostate documents, including incorrect survey meter data and omissions in quality checklists. Although the current version of the tool lacks image‐reading capabilities, it substantially improved the accuracy and speed of document validation. This tool reduces the burden of manual checks and ensures higher data quality and consistency.

**TABLE 3:** Chart check items listed in the LDR prostate brachytherapy monthly audit, with the ratio of checks that can be automated to total checks, and ineligible check items in our institution.

Monthly audit list	No. of documents that can checked by our scripts/No. of total documents	Ineligible check items that require manual inspection
Physician approval	2/4	Physician approvals in PDFs of OR plan and post plan, which were converted to PNG images in Aria, even though the original PDF plans can be automatically checked.
Physicist signatures	5/5	None.
OR plan and post plan done	0/2	All the information of these PDF reports is imported into Aria EMR as PNG images
Survey report, quality checklist, and seed order documents	2/3	PDF screenshot of confirmation from seed vendor in the seed order
Paperwork completed and filed (binder or electronic brachy log)	10/12	These paper files in the binder are not digitalized yet.
Shipment and assay documentation, log and inventories	0/1	Information is recorded in a log book which is not digitalized yet.

## 4 DISCUSSION

This study introduces an in‐house automation tool for verifying documents in LDR prostate brachytherapy, providing efficiency benefits compared to manual checks. Basic, rule‐based processes can streamline routine tasks in various applications. We highlight the benefits of using automation for specific clinical tasks like seed ordering and monthly audits, emphasizing time savings, and error detection before human intervention. These results underscore the importance of developing automation for LDR prostate brachytherapy, leveraging the spectrum of advantages observed in other studies for EBRT and HDR brachytherapy. Using our in‐house scripts with complementary tools for treatment planning and documentation in the VariSeed treatment planning system framework offers a higher level of automation potential, which may extend to other digital document applications. Drawing from previous research^8^ on escalating benefits with automation levels, we anticipate analogous efficiency gains and error reduction in LDR prostate brachytherapy, in parallel with technological advancements such as artificial intelligence.^17^, ^18^

Operating either independently or integrated into existing systems, our scripts enhance the breadth of assessments beyond conventional manual chart reviews for LDR brachytherapy, without direct intervention from physicists. An example is comparing the same data points in the quality check reports with survey reports and EOT documents. Our scripts' analysis revealed two inconsistencies in implanted seed counts across these documents, highlighting a potential issue for post‐CT planning. In integration mode, the scripts can be incorporated into our middleware and other software to further broaden the scope of assessments, such as EBRT documents and Care path status. Through the examination of a broader spectrum of data points, our methodology enables the swift identification of discrepancies or potential human errors, averting prolonged periods of oversight. Plus, the scripts demonstrate versatility by supporting data mining efforts for patient records and research endeavors, thereby highlighting its role in enhancing operational precision and efficiency. Our tool is adaptable to other EMR systems if documents can be exported manually or retrieved automatically in Word or PDF formats. However, certain automation of document retrieval might be limited depending on how convenient to query an EMR system and retrieve documents programmatically.

Automation approaches, while effective in reducing human errors and expediting processes, still cannot fully replace manual checks.^7^ Human intervention in decision‐making and exception handling remains crucial, emphasizing the need for vigilant monitoring and maintenance to ensure accurate and ethical automation use. For example, in the seed ordering process, we uphold the verification functions of our automated tool while recognizing the significance of physician discretion. Although developing a function to automatically calculate seed number for physicians may seem sufficient, it is important to note that physicians consider diverse factors such as treatment goals, patient characteristics, and treatment history. Hence, we strive to strike a balance between automation and clinical expertise in practical scenarios.

A limitation of our study lies in image recognition and information retrieval from digital images. Certain checklist items, like treatment reports embedded as PNG images in Aria Word documents, cannot be processed by our automated scripts. While our current method can analyze text within original PDFs, it falls short in automatically interpreting images or detecting upload‐related issues within the Aria EMR system. Therefore, one of our forthcoming research objectives is to refine the EMR document design for better script analysis or implement more robust scripting capabilities. Additionally, we could not conduct a Failure Mode and Effects Analysis (FMEA) following TG 100 guidelines^19^ due to limited relevant literature in the context of LDR brachytherapy. Chadwick et al's study,^20^ which introduced an approach similar to FMEA, primarily focuses on procedural aspects beyond our documentation verification responsibilities. As part of our future work, we also aim to develop a comprehensive FMEA tailored specifically to the unique requirements of LDR brachytherapy.

Our Python scripts are available to share upon request. For those interested in developing their tools for document verification, here are our recommendations: begin by setting clear objectives for what automation you intend to accomplish. Conduct comprehensive reviews of your current clinical workflow, checklists, and RO‐IS. Design and build your tool to access data specific to your workflow, prioritizing user‐friendliness. Validate and refine the tool with insights from past cases and feedback from pilot users. Train additional clinical staff for implementation and continuously optimize your tool to address gaps across different scenarios. Always understand the strengths and limitations of your tool.

## 5 CONCLUSIONS

This study serves as a pilot investigation into the automation of EMR document verification for LDR brachytherapy. Our findings affirm the efficiency enhancement of employing an automated tool for electronic document management, including routine chart checks, seed order monthly audits, and more.

## AUTHOR CONTRIBUTIONS

Huijun Xu conceived the study idea. Baoshe Zhang and Huijun Xu contributed to the study design and software development. Junliang Xu, Baoshe Zhang, and Huijun Xu drafted the manuscript and conducted the literature search. Mariana Guerrero and Huijun Xu collected the data for analysis. Junliang Xu, Chaitanya Kalavagunta, and Shifeng Chen edited the manuscript. All authors reviewed and approved the final version of the manuscript.

## CONFLICT OF INTEREST STATEMENT

The authors declare no conflict of interest.
